# Assessing the hidden burden and costs of COVID-19 pandemic in South Asia: Implications for health and well-being of women, children and adolescents

**DOI:** 10.1371/journal.pgph.0001567

**Published:** 2023-04-12

**Authors:** Aatekah Owais, Arjumand Rizvi, Muhammad Jawwad, Susan Horton, Jai K. Das, Catherine Merritt, Ralfh Moreno, Atnafu G. Asfaw, Paul Rutter, Phuong H. Nguyen, Purnima Menon, Zulfiqar A. Bhutta

**Affiliations:** 1 Centre for Global Child Health, Hospital for Sick Children, Toronto, Canada; 2 Division of Women and Child Health, Aga Khan University, Karachi, Pakistan; 3 School of Public Health and Health Systems, University of Waterloo, Waterloo, Canada; 4 Institute of Global Health and Development, The Aga Khan University, South-Central Asia, East Africa & United Kingdom; 5 UNICEF Headquarters, New York, New York, United States of America; 6 UNICEF ROSA, Kathmandu, Nepal; 7 International Food Policy Research Institute, Delhi, India; 8 Center of Excellence in Women and Child Health, The Aga Khan University, South-Central Asia, East Africa & United Kingdom; McGill University, CANADA

## Abstract

The COVID-19 pandemic has disproportionately affected vulnerable populations. With its intensity expected to be cyclical over the foreseeable future, and much of the impact estimates still modeled, it is imperative that we accurately assess the impact to date, to help with the process of targeted rebuilding of services. We collected data from administrative health information systems in six South Asian countries (Afghanistan, Bangladesh, Nepal, India, Pakistan and Sri Lanka), to determine essential health services coverage disruptions between January–December 2020, and January–June 2021, compared to the same calendar months in 2019, and estimated the impact of this disruption on maternal and child mortality using the Lives Saved Tool. We also modelled impact of prolonged school closures on continued enrollment, as well as potential sequelae for the cohort of girls who have likely dropped out. Coverage of key maternal and child health interventions, including antenatal care and immunizations, decreased by up to 60%, with the largest disruptions observed between April and June 2020. This was followed by a period of recovery from July 2020 to March 2021, but a reversal of most of these gains in April/May 2021, likely due to the delta variant-fueled surge in South Asia at the same time. We estimated that disruption of essential health services between January 2020 and June 2021 potentially resulted in an additional 19,000 maternal and 317,000 child deaths, an increase of 19% and 13% respectively, compared to 2019. Extended school closures likely resulted in 9 million adolescents dropping out permanently, with 40% likely being from poorest households, resulting in decreased lifetime earnings. A projected increase in early marriages for girls who dropped out could result in an additional 500,000 adolescent pregnancies, 153,000 low birthweight births, and 27,000 additional children becoming stunted by age two years. To date, the increase in maternal and child mortality due to health services disruption has likely exceeded the overall number of COVID-19 deaths in South Asia. The indirect effects of the pandemic were disproportionately borne by the most vulnerable populations, and effects are likely to be long-lasting, permanent and in some cases inter-generational, unless policies aimed at alleviating these impacts are instituted at scale and targeted to reach the poorest of the poor. There are also implications for future pandemic preparedness.

## Introduction

In the three years since COVID-19 was first identified in Wuhan, China, the pandemic continues to upend lives globally, reducing access to essential healthcare services and disrupting manufacturing and service industries. The stringent lockdowns during the first phase resulted in catastrophic economic fallout, with India’s Gross Domestic Product (GDP) per capita shrinking by almost 7% in 2020 [1, 2], leaving limited political appetite for similar sweeping measures thereafter. The development of variants of concern, such as delta and omicron leading to rapid upsurges in cases and continued deaths across the world, adds a level of uncertainty as to the duration of the pandemic, with wide variations in epidemiological patterns [3].

Globally, direct reports of COVID-19 mortality are likely to under-estimate true burden by over three folds [4]. In addition to the direct effects of COVID-19 in terms of morbidity and mortality, the impact of the pandemic response on population health and nutrition has been disproportionately borne by vulnerable populations. Evidence from past crises, such as the 1997 East Asian financial crisis, the 2008 global financial and food price increase crises, and the 2013–16 Ebola outbreak in West Africa, underscore the vulnerability of women and children, through reduced access to antenatal care, immunizations and schooling [5–10]. Evidence from the COVID-19 pandemic paints a similar picture, including deferral or non-receipt of antenatal care visits due to lockdowns and lack of transport, decrease in facility-based deliveries, and child growth monitoring and immunizations by up to 50%, especially in the early months of the pandemic [11–16].

In addition to severe health systems disruption, and virtual collapse in some settings, following the severe early stringent measures imposed by many countries, the consequences of interruption to essential services for sexual, reproductive, maternal and child health services (SRMNCH) are of increasing concern. It is anticipated that reduced access to family planning services in low- and middle-income countries (LMICs) could lead to millions of unintended pregnancies in the near future [17]. Early estimates, based on modelled disruptions to health services provision and access, and rising food insecurity ranging in severity, predicted an additional 253,500–1,157,000 child deaths and 12,200–56,700 maternal deaths, within one year [18]. Since then, several studies have assessed the actual impact of the COVID-19 pandemic on coverage of essential SRMNCH services [19–24]. However, most of the studies in LMICs have been carried out in smaller settings, such as health facilities or research cohorts, or included data collected very early in the pandemic.

We assessed disruption in coverage of essential healthcare services resulting from large-scale and rolling lockdowns implemented throughout 2020 and 2021 to curb the spread of COVID-19 in the six most populous countries in South Asia (Afghanistan, Bangladesh, Nepal, India, Pakistan and Sri Lanka), using actual public sector health services utilization data from January 2019 to June 2021. We also quantified the potential impact on maternal and child mortality, and unplanned pregnancies, as well as the impact on school enrollment and sequelae for girls who have likely dropped out of school due to prolonged closures.

## Methods

### Disruption of essential health services

We collaborated with the UNICEF Regional Office for South Asia (ROSA) who coordinated the data collation, mainly through UNICEF Country Office focal persons for routine services via existing Health Management Information System (HMIS) dashboards and/or through Ministry of Health focal persons in Afghanistan, Bangladesh, Sri Lanka, Nepal and Pakistan. Additionally, UNFPA and WHO Country Office focal persons also supported data extraction/collation from HMIS at country level as required. In India, colleagues from IFPRI collated data publicly available from HMIS, from January 2019 to May 2021.

Using the HMIS data received, we compiled country-specific datasets for essential SRMNCH service utilization from January 2019 to June 2021, computing quarterly values by summing up the raw data for monthly service utilization for each quarter. We assumed that the population eligible for receiving each service (i.e. denominator) would not have changed over the course of the study period. Disruption in service utilization between January 2019 and June 2021 was assessed by comparing changes in the raw data collected (i.e., numerator) for each essential SRMNCH service as follows:

- 2020 Q1 disruption: Change between Jan–Mar 2019 and Jan–Mar 2020
- 2020 Q2 disruption: Change between Apr–Jun 2019 and Apr–Jun 2020
- 2020 Q3 disruption: Change between Jul–Sep 2019 and Jul–Sep 2020
- 2020 Q4 disruption: Change between Oct–Dec 2019 and Oct–Dec 2020
- 2021 Q1 disruption: Change between Jan–Mar 2019 and Jan–Mar 2021
- 2021 Q2 disruption: Change between Apr–Jun 2019 and Apr–Jun 2021

Health systems data were not released for care-seeking for pneumonia and diarrhea, and treatment for severe acute malnutrition in Sri Lanka. Data on treatment for severe acute malnutrition for Nepal were also not released. For these, average service disruption estimates from all other countries were used as proxy. HMIS data from India were also not released for June 2021. We therefore used data from April and May 2021 to estimate coverage disruption in Q2 2021.

LiST does not provide confidence limits or any other analysis of uncertainly around the estimates it generates. To overcome this limitation, we used monthly DHIS data from 150+ districts in Pakistan to estimate health services utilization for each district from January 2019 to June 2021. The pre-pandemic values (January to December 2019) were compared with the post-pandemic values (January 2020 to December 2020 and January 2021 to June 2021), to estimate the change due to pandemic restrictions for each month in each district. We then used this dataset to estimate the median and 95% confidence intervals (CIs) for each essential SRMNCH service. We then ran three LiST models for each quarter, using the median, upper bound and lower bound, to calculate 95% CIs around the main effect. We were unable to carry out similar analyses for the other five countries, since we only had access to monthly or quarterly changes in health services utilizations at the national level during the study period, which did not provide adequate variability in the data to calculate CIs around the estimates.

### Maternal and child mortality

Given the lack of vital registration systems for mortality in the region, we used the Lives Saved Tool (LiST) [25], and the Family Planning (FamPlan) [25] modules of Spectrum [26], to estimate the increase in under-5 child and maternal mortality, as well as the increase in unplanned pregnancies among women aged 15–49 years, resulting from reduced access and provision of essential SRMNCH services. LiST is a deterministic, linear mathematical model, which describes fixed relationships between inputs and outputs, producing the same output, given the same set of inputs, each time [25]. The primary inputs included in LiST are coverage of maternal and child health interventions, such as antenatal care, immunizations, and treatment for diarrhea and pneumonia [25]. The primary outputs include population-level indicators of maternal and child morbidity and mortality [25]. LiST allows for the impact estimation of change in coverage of multiple interventions, simultaneously [25]. Information on coverage and effectiveness of interventions included in LiST is available here: https://www.livessavedtool.org/about-1.

For our analysis, inputs included coverage of essential SRMNCH services and outputs include cause-specific maternal and child mortality. Changes in outputs are estimated based on specified change in coverage of inputs. Additionally, LiST provides yearly estimates. To generate quarterly estimates, we developed separate projections for each country for every quarter and divided the estimates by 4 to get quarterly estimates. Details of the theoretical approach and basic modeling structure used in LiST are available elsewhere [25]. We mapped indicators available from HMIS with those included in LiST. The list of indicators used in our LiST analysis for each country are included in the Table A in S1 Text.

### Educational attainment of school-aged children

We assessed the potential impact of the COVID-19 pandemic on educational attainment of school-aged children in six South Asian countries, and its sequelae on individual earnings and national GDPs. Loss in educational attainment can potentially occur in multiple ways, such as loss of learning time or loss of already acquired learning due to school closures [27]. However, we focused on the loss of educational attainment that was likely to occur due to the increase in number of students who permanently drop out of school because of prolonged school closures.

We estimated the current size of the cohort of children enrolled in primary and secondary schools using population estimates available from UNESCO [28], and net attendance ratios available from the most recent DHS, for each country [29–33]. We used age- and quintile-specific school dropout rates, adapted from those observed during the 1997 East Asian financial crisis in Indonesia (Table B in S1 Text) [34].

We assumed that those who dropped out in primary school would on average complete a maximum of 2·5 years, and those who drop out in secondary school would complete about 8·5 years of education. Primary school normally lasts for 4 years and secondary school an additional 8 years in this region. The corresponding years of education lost for each country were calculated based on the highest median years of schooling completed, irrespective of age and gender (Table C in S1 Text). We estimated income loss associated with reduced educational attainment by assuming that one less year of primary and secondary education reduces an individual’s lifetime income by 4·04% and 2·44%, respectively [35]. The 2019 GDP per capita for each country was assumed as baseline. We assumed country-specific wage growth over an individual’s economic lifetime (i.e. 45 years) to be the same as the estimated GDP growth rate in 2021 [36–41]. A discount rate of 3% was applied to calculate the present value of loss in lifetime earnings.

### Early marriage and adolescent pregnancies

We also estimated the expected number of girls who are likely to drop out of school as a result of the pandemic, using gender-specific school dropout rates observed during the 1997 East Asian financial crisis in Indonesia [34]. Dropping out of school is associated with early marriage among girls in South Asia [42].

We estimated the number of adolescent pregnancies likely to occur for this cohort of girls. We used the baseline prevalence of adolescent pregnancies reported in the most recent DHS for each country [29–33, 43], and assumed that adolescent pregnancy rates could increase by 10–15% over the next few years, based on recent evidence from Bangladesh [44], as well as the experiences of Syrian refugees in Jordan [45] and Lebanon [46]. We assumed that although risk of maternal mortality in adolescent pregnancies would likely be the same as those observed for women > 19 years [47], risk of neonatal mortality and low birthweight births could increase by 22.5% and 19%, respectively, based on the existing evidence of excessive risks among young mothers [48]. We conservatively assumed that at least 20% of children born with low birthweight were likely to be stunted by age two years [49, 50].

## Results

### Disruption of essential health services

**Fig 1:**
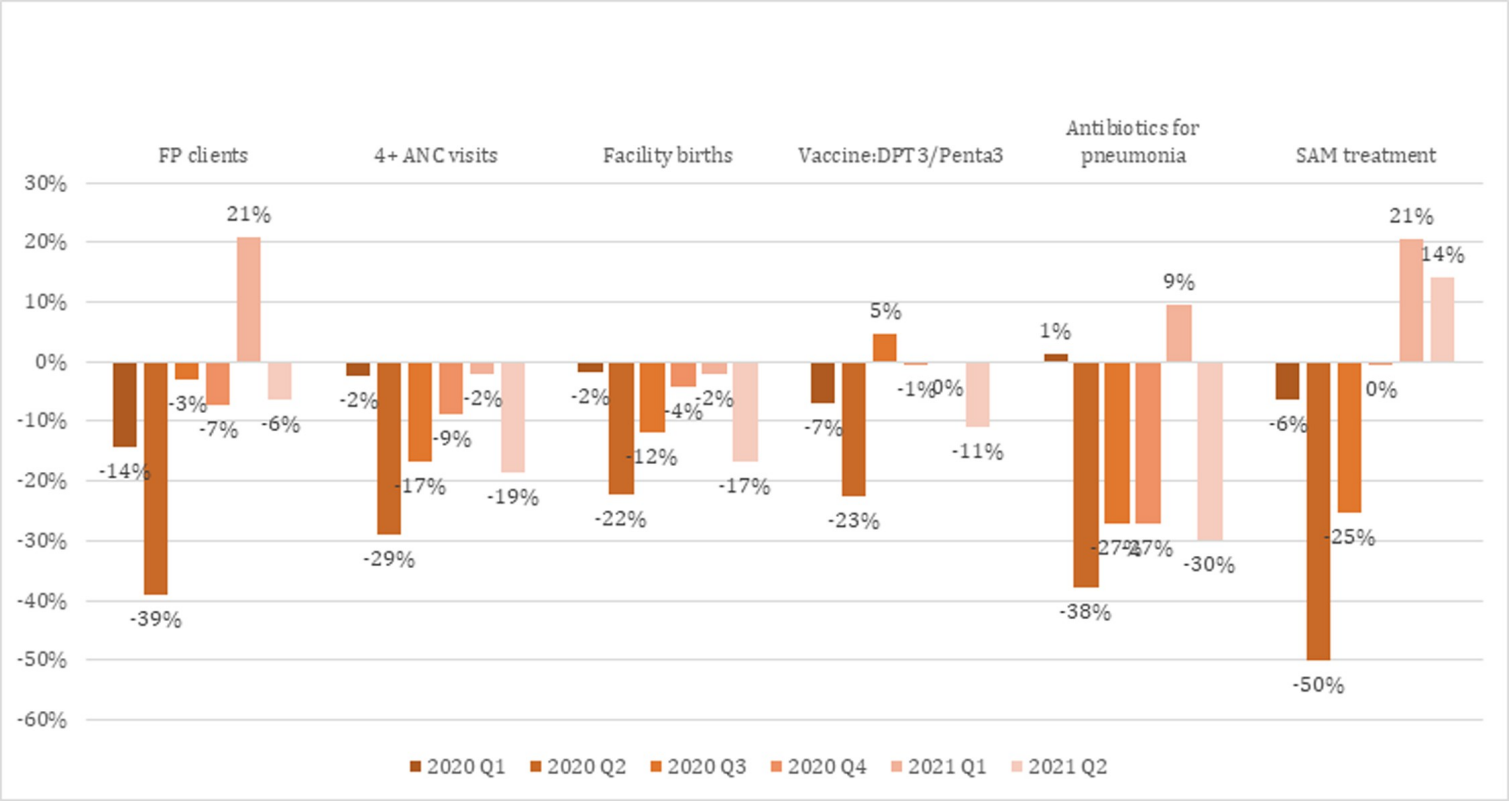
Observed disruption in utilization of essential SRMNCH health services due to COVID-19 in South Asia, Jan 2020 to Jun 2021.

Even before the WHO declared COVID-19 as a pandemic, coverage of essential, SRMNCH services were being affected in several countries in South Asia. Based on data collected from country health information systems, coverage of family planning services decreased by 1–32% in five of the six South Asian countries included in our analysis in the first quarter of 2020, compared to what was observed during the same period in 2019. Afghanistan is the only country that reported a 7% increase in coverage of family planning services over this period, which could be questioned as anomalous (Table 1). In the second quarter of 2020, which corresponds with when the most stringent COVID-19 control strategies were instituted in the region, coverage of essential SRMNCH services declined substantially in South Asia (Fig 1). However, there were variation by country. Bangladesh and Pakistan observed the most disruption, while Sri Lanka observed the least. Coverage disruption of selected SRMNCH services observed in each country in 2020 are presented in Table 1, with detailed results included in Table D in S1 Text.

**Table 1:** Observed coverage disruption of selected SRMNCH services due to the COVID-19 pandemic in South Asia, Jan 2020 to Jun 2021.

	Pakistan						Afghanistan					
Interventions	Change in 2020				Change in 2021		Change in 2020				Change in 2021	
	**Q1**	**Q2**	**Q3**	**Q4**	**Q1**	**Q2**	**Q1**	**Q2**	**Q3**	**Q4**	**Q1**	**Q2**
FP services	-31.8%	-71.7%	-35.4%	-14.1%	-22.8%	-33.1%	7.3%	-17.9%	-18.2%	-6.8%	145.0%	115.0%
4+ ANC visits	-16.7%	-56.2%	-27.9%	-4.7%	-28.7%	-29.7%	0.1%	-14.4%	2.2%	-2.8%	9.0%	4.6%
Facility births	0.0%	-36.7%	-24.9%	-3.3%	-14.5%	-28.8%	4.4%	-0.4%	-0.7%	0.9%	12.8%	16.0%
Vaccine: DPT3/Penta3	-22.4%	-55.1%	-26.7%	-10.2%	-28.8%	-22.1%	6.6%	-9.5%	13.4%	4.5%	7.9%	1.2%
Antibiotics for pneumonia	-10.3%	-69.0%	-43.4%	-29.5%	-38.5%	-41.7%	15.2%	-8.6%	-0.4%	-2.7%	5.5%	7.9%
SAM treatment	32.2%	-60.8%	-37.0%	-8.1%	-34.2%	-43.6%	4.6%	-37.4%	-19.0%	-29.2%	36.0%	54.0%
	**Bangladesh**						**Nepal**					
**Interventions**	**Change in 2020**				**Change in 2021**		**Change in 2020**				**Change in 2021**	
	**Q1**	**Q2**	**Q3**	**Q4**	**Q1**	**Q2**	**Q1**	**Q2**	**Q3**	**Q4**	**Q1**	**Q2**
FP services	-21.4%	-62.0%	-37.6%	-28.0%	-3.4%	-22.2%	-22.3%	-51.9%	57.9%	-5.3%	-1.3%	-42.9%
4+ ANC visits	6.7%	-45.1%	-48.3%	-36.3%	-18.5%	-26.4%	-9.3%	-29.2%	-14.7%	-5.6%	20.5%	-15.4%
Facility births	-7.0%	-42.0%	-29.8%	-15.5%	-13.3%	-27.2%	-6.6%	-26.9%	-8.3%	-4.9%	2.5%	-13.1%
Vaccine: DPT3/Penta3	-6.0%	-36.7%	20.1%	-1.5%	9.2%	5.9%	-22.7%	3.0%	19.7%	1.5%	6.3%	-6.0%
Antibiotics for pneumonia	-13.2%	-71.3%	-55.2%	-27.5%	-3.9%	-23.7%	-11.5%	-48.7%	-54.7%	-56.3%	-37.6%	-53.7%
SAM treatment	-27.0%	-70.5%	-27.2%	59.3%	103.0%	100.7%	-53.1%	-83.4%	-53.2%	-34.0%		
	**Sri Lanka**						**India**					
**Interventions**	**Change in 2020**				**Change in 2021**		**Change in 2020**				**Change in 2021**	
	**Q1**	**Q2**	**Q3**	**Q4**	**Q1**	**Q2**	**Q1**	**Q2**	**Q3**	**Q4**	**Q1**	Q2*
FP services	-17.3%	-6.6%	18.7%	7.1%	2.5%	-17.2%	-0.8%	-25.0%	-3.2%	3.6%	4.7%	-38.6%
4+ ANC visits	0.0%	-2.1%	-2.5%	-1.9%	-2.3%	-2.6%	4.7%	-26.7%	-8.5%	-1.0%	7.4%	-41.5%
Facility births	0.0%	0.0%	0.0%	0.0%	0.0%	0.0%	-2.1%	-28.2%	-7.5%	-2.6%	-0.7%	-46.7%
Vaccine: DPT3/Penta3	No data received						2.8%	-37.0%	1.0%	1.9%	4.8%	-44.0%
Antibiotics for pneumonia							27.4%	-29.9%	-9.4%	-46.6%	131.3%	-67.9%
SAM treatment							5.1%	-48.2%	-14.9%	9.6%	18.4%	-26.7%

FP: Family planning; ANC: Antenatal care; SAM: Severe acute malnutrition.

*April and May 2021 data used for Q2 2021.

From July 2020 to March 2021, there was a gradual recovery, with coverage of DPT3/Penta3 immunization improving by 5% in Q3 of 2020, most likely due to catch-up vaccination drives (Fig 1), but coverage dipped below pre-pandemic levels in Q4 of 2020. At the end of 2020, coverage of all essential SRMNCH services remained below or at pre-pandemic level in South Asia, compared to the same period in 2019.

In Q1 of 2021 coverage of family planning services, treatment for pneumonia and severe acute malnutrition among children under-five increased substantially to above pre-pandemic levels. All other services had also recovered to, or nearly to pre-pandemic levels. However, these gains were reversed by Q2 of 2021, which coincided with the delta variant-fueled surge in India and the rest of South Asia in April and May 2021.

### Maternal and child mortality

The disruption in SMRNCH services has likely had a substantial impact on maternal and child mortality in South Asia. Table 2 summarizes the estimated increase in maternal and under-five child mortality, and pregnancies for each country by each quarter between Jan 2020 and Jun 2021. The number of deaths among children under-five are estimated to have increased by more than 317,000 across the six South Asian countries in this period compared to 2019, with more than 150,000 of these deaths occurring in the neonatal period. The greatest increases were estimated for India (15% increase) and Pakistan (12% increase), respectively. For all countries, the largest increase in deaths is estimated for April–June 2020.

**Table 2:** Estimated increase in deaths and unintended pregnancies by country, in South Asia, Jan 2020 to Jun 2021.

	2020/21	Afghanistan	Bangladesh	India	Nepal	Pakistan	Sri Lanka	Overall
Child deaths	Q1	-3.5%	3.9%	0.0%	6.2%	2.3%	1.2%	0.8%
	Q2	2.8%	25.5%	39.3%	12.9%	26.7%	3.9%	33.1%
	Q3	1.2%	9.9%	8.8%	1.9%	16.9%	-0.4%	10.4%
	Q4	1.2%	6.1%	9.7%	2.6%	5.0%	-0.3%	7.8%
	Q1	-4.5%	-2.4%	-12.5%	-1.2%	6.1%	-0.5%	-6.7%
	Q2	-5.1%	1.7%	45.3%	8.7%	13.1%	1.0%	31.9%
	**Overall**	-1.3%	7.5%	15.1%	5.2%	11.7%	0.8%	12.9%
Neonatal deaths	Q1	-4.5%	2.6%	2.2%	2.6%	0.3%	0.7%	1.4%
	Q2	2.2%	17.2%	36.5%	16.6%	30.5%	1.0%	32.0%
	Q3	1.8%	11.4%	10.7%	0.2%	20.8%	-1.1%	12.7%
	Q4	1.1%	5.7%	3.5%	-0.6%	4.1%	-0.7%	3.6%
	Q1	-6.8%	0.9%	-3.0%	-0.9%	6.3%	0.1%	-0.6%
	Q2	-7.9%	5.5%	53.7%	12.5%	18.7%	1.3%	38.7%
	**Overall**	-2.4%	7.2%	17.3%	5.1%	13.5%	0.2%	14.6%
Stillbirths	Q1	-6.7%	0.6%	1.1%	1.1%	0.3%	0.5%	0.6%
	Q2	1.6%	6.9%	26.7%	14.1%	21.0%	0.7%	22.7%
	Q3	1.6%	2.6%	8.4%	-0.6%	17.1%	-1.4%	9.8%
	Q4	1.2%	1.3%	2.3%	0.2%	2.2%	-1.0%	2.1%
	Q1	-7.4%	-1.1%	-2.3%	-1.3%	2.9%	-0.4%	-1.0%
	Q2	-7.9%	0.2%	42.7%	12.7%	15.8%	1.0%	30.4%
	**Overall**	-2.9%	1.7%	13.2%	4.3%	9.9%	-0.1%	10.8%
Maternal deaths	Q1	-15.5%	0.3%	-1.6%	6.1%	1.2%	2.2%	-1.8%
	Q2	3.3%	21.3%	47.1%	34.4%	40.5%	2.2%	40.6%
	Q3	1.6%	11.8%	18.6%	0.1%	30.6%	-1.1%	18.2%
	Q4	1.4%	7.5%	7.3%	1.8%	3.4%	-1.1%	6.2%
	Q1	-19.6%	-3.6%	-8.1%	-4.2%	6.5%	1.1%	-6.3%
	Q2	-19.9%	-0.3%	76.5%	25.2%	28.1%	3.3%	55.5%
	**Overall**	-8.1%	6.2%	23.3%	10.6%	18.4%	1.1%	18.7%
Unintended pregnancies	Q1	-1.2%	2.1%	0.3%	1.0%	1.8%	4.3%	0.7%
	Q2	0.8%	5.8%	39.8%	5.3%	16.1%	4.5%	31.0%
	Q3	0.8%	2.9%	16.3%	-4.6%	7.8%	-1.9%	12.7%
	Q4	-0.1%	1.9%	3.8%	-0.4%	7.7%	-0.7%	3.8%
	Q1	-2.9%	-1.6%	-3.7%	0.7%	2.2%	0.8%	-2.6%
	Q2	-2.2%	-0.5%	31.1%	6.0%	5.2%	5.0%	22.7%
	**Overall**	-0.8%	1.8%	14.6%	1.3%	6.8%	2.0%	11.4%

The number of stillbirths is also estimated to have gone up in the region. Across South Asia as a whole, an estimated 150,000 additional stillbirths are likely to have occurred by June 2021 because of reduced coverage of essential SRMNCH services. At the country-level, the largest increase in the number of stillbirths is expected to have occurred in India (13% increase), followed by Pakistan (10% increase), Nepal (4% increase) and Bangladesh (2% increase).

Similarly, the number of maternal deaths is also expected to have increased between Jan 2020 and Jun 2021 as a result of the COVID-19 pandemic response, compared to those observed in 2019, with the highest number of additional deaths anticipated in India (23% increase) and Pakistan (18% increase). Due to the observed reduction in coverage of modern contraceptive methods, more than 5.4 million additional unintended pregnancies have potentially occurred in South Asia, with the highest number likely in India (approximately 5.0 million).

As mentioned previously, we were only able to calculate 95% CIs around estimates of disruptions in essential SRMNCH services and corresponding LiST estimates for Pakistan only (Tables E and F in S1 Text, respectively). These results indicate that the impact of pandemic-related restrictions on maternal and child morbidity and mortality was highly significant. Overall, in South Asia, maternal and child mortality is expected to have increased by 19% and 13%, respectively. These indirect effects on maternal and child mortality are far in excess of any potential direct deaths related to COVID-19 infections.

### Educational attainment of school-aged children

South Asia is home to approximately 420 million school-aged children, many of whom have been out of school since March 2020. As a result of prolonged school closures in response to the COVID-19 pandemic 9.4 million primary and secondary school-aged children are expected to permanently drop out of schools, with the highest number expected in India (approximately 7.5 million) (Table G in S1 Text).

The disruption in education is also expected to have considerable economic costs over the long term. Across South Asia, lower educational attainment by this cohort will result in a 15–30% decrease in their future lifetime earnings. The highest cost is expected to be borne by children in Sri Lanka (30% decrease), followed by India (24% decrease) and Nepal (20% decrease).

Less wealthy households are likely to experience the worst impact of school closures on educational attainment, and the consequent impact on lifetime earnings. Among those who will likely drop out of school permanently, 29–50% will be children from households in poorest wealth quintile, leading to increased income inequality over their lifetime. The estimated number of children expected to permanently drop out of school, and the impact this will have over the course of these children’s lifetimes is summarized in the Tables G and H in S1 Text.

### Early marriage and adolescent pregnancies

Of the 9.4 million children expected to permanently drop out of school, 4·5 million of them are expected to be girls (Table 3). Given the cultural and social context of South Asia, as well as the economic hardship many families in the region are facing because of the COVID-19 pandemic and response, many of these girls are likely to be married off early. As a result, for this cohort of girls, the number of adolescent pregnancies is expected to increase by almost 500,000 in the region, and could lead to an additional 800 maternal and more than 13,000 neonatal deaths, 150,000 low birthweight births, and 27,000 children who are likely to be stunted by the age of two years. Table 3 summarizes the number of girls expected to permanently dropout of school and the maternal and child health-related sequelae resulting from early marriages and consequent adolescent pregnancies.

**Table 3:** Estimated number of additional adolescent pregnancies, maternal and neonatal deaths, low birthweight births and stunted children resulting from girls dropping out of school due to the COVID-19 pandemic in six South Asian countries.

Country	School dropouts	Adolescent pregnancies	Maternal deaths	Neonatal deaths	LBW births	Stunted
Afghanistan	69,288	9,222–9,641	59–62	418–437	2,964–3,098	510–533
Bangladesh	352,250	107,331–112,209	186–194	2,236–2,337	35,763–37,389	6,706–7,011
India	3,531,683	306,903–320,853	445–465	8,647–9,041	98,609–103,091	17,993–18,811
Nepal	84,587	15,539–16,245	29–30	381–399	4,069–4,253	738–771
Pakistan	397,453	35,413–37,023	50–52	1,823–1,905	11,379–11,896	1,912–1,999
Sri Lanka	66,024	2,179–2,278	1	11–12	415–434	81–85
**Total**	**4,501,285**	**476,587–498,250**	**769–804**	13,514–14,128	153,195–160,159	27,937–29,207

## Discussion

Our findings from country health information systems show that even by March 2021, countries in South Asia had not recovered fully from the disruptions in essential health services experienced during the first phase of the COVID-19 pandemic. Following the initial set of the delta variant-fueled surge in April and May of 2021, another wave of disruptions was triggered in essential SRMNCH services in South Asia, which substantially set back coverage recovery since July 2020. To our knowledge, this is the first systematic study from South Asia to assess the disruption in services across a large population (> 1·5 billion) and show that the effects continued to be felt well into the second year of the pandemic. Although South Asian countries have done reasonably well with COVID-19 vaccination rates so far [51], the vast inequities in global COVID-19 vaccine rollout across LMICs, and the risk of variants emerging and leading to renewed surges and continuation of the pandemic remains.

Regardless, there are major lessons learnt from the experience of the current pandemic. Our study highlights the need for continued provision of essential SRMNCH services and keeping schools open during future epidemics/pandemics. To date, studies have shown negligible risks to children or increased risk of community spread of COVID-19 when schools are open [52–54]. Furthermore, the estimated increase in child mortality due to COVID-19 related restrictions in our analysis far outweighs the number of children estimated to have died due to COVID-19 itself [55].

Our findings underscore the critical and continued need to address the much larger and longer-term fallout from the indirect effects of the pandemic. According to our estimates, an additional ~317,000 child, and ~19,000 maternal deaths potentially occurred in South Asia by June 2021. As estimated in Nepal, between March and May 2020, institutional stillbirth rates likely increased by 50% and neonatal mortality rate increased three-folds [20]. Additionally, between 2020 and 2021, all-cause excess mortality in South Asia is estimated to be more than 9-fold the reported deaths due to COVID-19 [4]. The fact that coverage for many key interventions continues to lag, is likely a reflection of population-level behaviors regarding care-seeking practices, as well as interruption of services. Even if coverage of essential services improves to pre-COVID-19 levels or better in 2022 and beyond, specific focus on overcoming gaps in immunizations and preventive services, such as antenatal and postnatal care, is needed to reach marginalized groups. A focus on strategies to address inequities and targeted implementation of interventions, such as reaching zero-dose children, would ensure that we emerge from the pandemic with stronger and more equitable systems. However, these strategies would need to account for country-specific contexts, with flexible and innovative implementation frameworks, bringing together individuals and organizations across a variety of expertise.

Education has been the major casualty of the COVID-19 pandemic, with over 90% of the world’s students forced to stay home during temporary school-closures in 2020 and 2021. South Asia has had the longest school closures across all of developing Asia, expected to result in an average loss of 0.49–0.65 learning-adjusted years of schooling [56]. Given the prolonged school closures, exacerbated by falling household incomes due to the economic shock experienced in the region, we estimate that more than nine million children potentially dropped out of school, with cascading impacts for the girls who likely left school permanently, including early marriage, adolescent pregnancies and sequelae. Although remote learning strategies were implemented in the region and could mitigate the extent of dropouts, 66% of the region’s population lives in rural areas, where access to remote learning is estimated to be less than 30% [57]. Recent data from Pakistan indicates that some 86% of children returned to school [58].

Although not assessed in the present study, the additional indirect effects of school closures on children’s mental health and wellbeing is now well recognized. These potential adverse effects were highlighted at an early stage of the pandemic [59], and have recently been confirmed through a systematic analysis of the impact of school closures on mental health and wellbeing of children, indicating a significant increase in anxiety and depression in school-aged children affected by closures [60].

Our analysis has several limitations, most if not all, resulting from constraints around data availability from health information systems, especially at sub-national level. Although we were able to use country-level health systems data for most indicators included in our LiST analysis, for coverage of childhood immunizations, care-seeking for pneumonia and diarrhea, and treatment for severe acute malnutrition in Sri Lanka we had to use average estimates from the other countries, as proxies. We also did not have access to full and complete HMIS data from each country (except Pakistan), which restricted our ability to produce confidence bounds around estimates. Furthermore, we were not given access to data from India for June 2021. Additionally, data quality issues for health administrative data are well established [61]. For e.g., in the India HMIS data, the number of women who received iron-containing supplements or four or more ANC visits was found to be larger than the number of pregnant women registered, and likely resulting in an overestimate of coverage and recovery of essential services. We also faced limitations with data availability on exact rates of school dropouts in South Asia, as these were not measured. We were therefore constrained to apply the assumptions on rates of school dropouts from Indonesia during the 1997 Asian financial crisis, across all six countries, but believe that these represent relatively conservative figures, given data from Pakistan [58]. Finally, our results are based on models estimating the impact of COVID-19 related restrictions on morbidity and mortality among women and children, and the increase and gendered impact of school dropouts, and are therefore approximations. The true extent of the indirect costs of the pandemic can only be ascertained by comprehensive, population-based surveys designed specifically to capture these data.

In summary, our findings and those of prior studies, confirm that the COVID-19 pandemic has had serious consequences for maternal and child health and education in South Asia [18, 20, 62]. All countries, including those in South Asia, need to continue, and even increase investments in health systems, poverty alleviation, education and creation of human capital, and gender equity, in order to maintain and improve on the gains made over the past few decades, especially during the Millennium Development Goals period. We need to do more than just catch up the loss in health and human capital experienced over the past two years. As suggested by Marmot and others [63], we need to build back better, to actively overcome gaps in equitable access and disadvantages faced by populations, simply because of the geographic region they live in.

These inequities are most apparent in the global rollout of COVID-19 immunizations. According to the Global Dashboard for Vaccine Equity, by April 2022, only 15% of people in low income countries had received at least 1 dose, compared to 72% in high income countries [64]. The emergence of more transmissible, and perhaps more virulent variants is likely a direct consequence of these inequities [65]. The only way out of the current pandemic, and mitigating the impact of the next one, is to ensure that strategies aimed at overcoming healthcare access and education related inequities, as well as efforts to protect the most vulnerable populations, remain at the forefront of every global public health response.

## Supporting information

S1 Text Supplementary tables. (DOCX)
