# Supplementary material for: Assessing the hidden burden and costs of COVID-19 pandemic in South Asia: Implications for health and well-being of women, children and adolescents
Source: PLOS Glob Public Health. 2023 Apr 12;3(4):e0001567. doi: 10.1371/journal.pgph.0001567 (PMC10096516; doi:10.1371/journal.pgph.0001567)
Supplement: S1 Text — (DOCX) [file pgph.0001567.s001.docx]

# Web Appendix

Table A: List of indicators used in LiST analysis for each country in South Asia

|  | **India** | | **Sri Lanka** | | **Nepal** | | **Bangladesh** | | **Afghanistan** | | **Pakistan** | |
| --- | --- | --- | --- | --- | --- | --- | --- | --- | --- | --- | --- | --- |
| **Interventions** | Health system data | Coverage | Health system data | Coverage | Health system data | Coverage | Health system data | Coverage | Health system data | Coverage | Health system data | Coverage |
| **Family Planning** |  |  |  |  |  |  |  |  |  |  |  |  |
| Injectables | ✓ | ✓ | ✓ | No DHS or MICS survey available. LiST default data pack was used | Proxy used: Average of IUD & implants | ✓ | Proxy used: Available FP services |  | Proxy used: Couple year of protection | ✓ | ✓ | ✓ |
| Condom | ✓ | ✓ | ✓ |  | Proxy used: Average of IUD & implants | ✓ | Proxy used: Available FP services |  | Proxy used: Couple year of protection | ✓ | ✓ | ✓ |
| IUD | ✓ | ✓ | ✓ |  | ✓ | ✓ | Proxy used: Available FP services |  | Proxy used: Couple year of protection | ✓ | ✓ | ✓ |
| Female sterilisation | ✓ | ✓ | ✓ |  | Proxy used: Average of IUD & implants | ✓ | Proxy used: Available FP services |  | Proxy used: Couple year of protection | ✓ | ✓ | ✓ |
| Male sterilisation | ✓ | ✓ | ✓ |  | Proxy used: Average of IUD & implants | ✓ | Proxy used: Available FP services |  | Proxy used: Couple year of protection | ✓ | ✓ | ✓ |
| Implant | ✓ | ✓ | ✓ |  | ✓ | ✓ | Proxy used: Available FP services |  | Proxy used: Couple year of protection | ✓ | ✓ | ✓ |
| **Periconceptual** |  |  |  |  |  |  |  |  |  |  |  |  |
| Folic acid supplementation | 🗶 | 🗶 | ✓ |  | 🗶 | 🗶 | 🗶 | 🗶 | 🗶 | 🗶 | 🗶 | 🗶 |
| Safe abortion services | ✓ | 🗶 | Proxy used: FP services |  | Proxy used: FP services | Regional defaults used | Proxy used: FP services | Regional defaults used | Proxy used: FP services | Regional defaults used | proxy used : FP services | Regional defaults used |
| Post-abortion case management | ✓ | 🗶 | 🗶 |  | 🗶 | 🗶 | 🗶 | 🗶 | 🗶 | 🗶 | 🗶 | 🗶 |
| Ectopic pregnancy case management | ✓ | 🗶 | 🗶 |  | 🗶 | 🗶 | 🗶 | 🗶 | 🗶 | 🗶 | 🗶 | 🗶 |
| **Pregnancy (routine care)** |  |  |  |  |  |  |  |  |  |  |  |  |
| Antenatal care (4+ visits) | ✓ | ✓ | Proxy used: PNC |  | ✓ | ✓ | ✓ | ✓ | ✓ | ✓ | ✓ | ✓ |
| Tetanus Toxoid (2 or more doses) | ✓ | ✓ | Proxy used: PNC |  | Proxy used: ANC4+ | ✓ | Proxy used: ANC4+ | ✓ | Proxy used: ANC4+ | ✓ | ✓ | ✓ |
| Syphilis detection and treatment | ✓ | Calculated | Proxy used: ANC4+ |  | Proxy used: ANC4+ | Calculated | Proxy used: ANC4+ | Calculated | Proxy used: ANC4+ | Calculated | Proxy used: ANC4+ | Calculated |
| **Nutrition** |  |  |  |  |  |  |  |  |  |  |  |  |
| Calcium supplementation | ✓ | 🗶 | 🗶 |  | 🗶 | 🗶 | 🗶 | 🗶 | 🗶 | 🗶 | 🗶 | 🗶 |
| Iron supplementation in pregnancy | ✓ | ✓ | Proxy used: ANC4+ |  | Proxy used: ANC4+ | ✓ | Proxy used: ANC4+ | ✓ | Proxy used: ANC4+ | ✓ | Proxy used: ANC4+ | ✓ |
| **Case Management** |  |  |  |  |  |  |  |  |  |  |  |  |
| Hypertensive disorder case management | ✓ | Calculated | Proxy used: ANC4+ |  | Proxy used: ANC4+ | Calculated | Proxy used: ANC4+ | Calculated | Proxy used: ANC4+ | Calculated | Proxy used: ANC4+ | Calculated |
| Diabetes case management | ✓ | Calculated | Proxy used: ANC4+ |  | Proxy used: ANC4+ | Calculated | Proxy used: ANC4+ | Calculated | Proxy used: ANC4+ | Calculated | Proxy used: ANC4+ | Calculated |
| Malaria case management | Proxy used: ANC4+ | Calculated | Proxy used: ANC4+ |  | Proxy used: ANC4+ | Calculated | Proxy used: ANC4+ | Calculated | Proxy used: ANC4+ | Calculated | Proxy used: ANC4+ | Calculated |
| MgSO4 management of pre-eclampsia | ✓ | Calculated | Proxy used: ANC4+ |  | Proxy used: ANC4+ | Calculated | Proxy used: ANC4+ | Calculated | Proxy used: ANC4+ | Calculated | Proxy used: ANC4+ | Calculated |
| **Other** |  |  |  |  |  |  |  |  |  |  |  |  |
| Fetal growth restriction detection and management | ✓ | 🗶 | 🗶 |  | 🗶 | 🗶 | 🗶 | 🗶 | 🗶 | 🗶 | 🗶 | 🗶 |
| **Childbirth** |  |  |  |  |  |  |  |  |  |  |  |  |
| Facility births | ✓ | ✓ | ✓ |  | ✓ | ✓ | ✓ | ✓ | ✓ | ✓ | ✓ | ✓ |
| **Preventive** |  |  |  |  |  |  |  |  |  |  |  |  |
| Postnatal visit within 2 days after birth | ✓ | ✓ | ✓ |  | ✓ | ✓ | ✓ | ✓ | Proxy used: Facility births | ✓ | ✓ | ✓ |
| Vitamin A supplementation* | ✓ | ✓ | ✓ |  | ✓ | ✓ | 🗶 | 🗶 | 🗶 | 🗶 | 🗶 | ✓ |
| Zinc Supplementation | ✓ | 🗶 | 🗶 |  | 🗶 | 🗶 | 🗶 | 🗶 | 🗶 | 🗶 | 🗶 | 🗶 |
| **Vaccines** |  |  |  |  |  |  |  |  |  |  |  |  |
| Vaccine: BCG | ✓ | ✓ | 🗶 |  | 🗶 | ✓ | 🗶 | ✓ | 🗶 | ✓ | 🗶 | ✓ |
| Vaccine: OPV3 | ✓ | ✓ | 🗶 |  | 🗶 | ✓ | 🗶 | ✓ | 🗶 | ✓ | 🗶 | ✓ |
| Vaccine: DPT3/Penta3 | ✓ | ✓ | Proxy used: Other country averages |  | ✓ | ✓ | ✓ | ✓ | ✓ | ✓ | ✓ | ✓ |
| Vaccine: HepB3 | ✓ | ✓ | Proxy used: Other country averages |  | Proxy used: Same as DPT3/Penta3 | ✓ | Proxy used: Same as DPT3/Penta3 | ✓ | Proxy used: Same as DPT3/Penta3 | ✓ | Proxy used: Same as DPT3/Penta3 | ✓ |
| Vaccine: PCV3 | ✓ | 🗶 | Proxy used: Other country averages |  | Proxy used: Same as DPT3/Penta3 | ✓ | Proxy used: Same as DPT3/Penta3 | ✓ | Proxy used: Same as DPT3/Penta3 | ✓ | Proxy used: Same as DPT3/Penta3 | ✓ |
| Vaccine: RV2 | ✓ | 🗶 | 🗶 |  | 🗶 | 🗶 | 🗶 | 🗶 | 🗶 | 🗶 | 🗶 | 🗶 |
| Vaccine: Measles | ✓ | ✓ | Proxy used: Other country averages |  | Proxy used: Same as DPT3/Penta3 | ✓ | Proxy used: Same as DPT3/Penta3 | ✓ | ✓ | ✓ | ✓ | ✓ |
| **Curative** |  |  |  |  |  |  |  |  |  |  |  |  |
| Maternal sepsis case management | ✓ | 🗶 | 🗶 |  | 🗶 | 🗶 | 🗶 | 🗶 | 🗶 | 🗶 | 🗶 | 🗶 |
| ORS - oral rehydration solution | ✓ | ✓ | 🗶 |  | ✓ | ✓ | ✓ | ✓ | ✓ | ✓ | ✓ | ✓ |
| Antibiotics for treatment of dysentery | ✓ | 🗶 | 🗶 |  | 🗶 | 🗶 | 🗶 | 🗶 | 🗶 | 🗶 | 🗶 | 🗶 |
| Zinc for treatment of diarrhea – Zinc Supplementation | ✓ | ✓ | Proxy used: Other country averages |  | ✓ | ✓ | ✓ | ✓ | ✓ | ✓ | ✓ | ✓ |
| Antibiotics for pneumonia | ✓ | 🗶 | Proxy used: Other country averages |  | ✓ | ✓ | ✓ | ✓ | ✓ | ✓ | ✓ | ✓ |
| Vitamin A for treatment of measles | ✓ | ✓ | 🗶 |  | 🗶 | 🗶 | 🗶 | 🗶 | 🗶 | 🗶 | 🗶 | 🗶 |
| ACTs - Artemisinin compounds for treatment of malaria | ✓ | 🗶 | 🗶 |  | 🗶 | 🗶 | 🗶 | 🗶 | 🗶 | 🗶 | 🗶 | 🗶 |
| SAM - treatment for severe acute malnutrition | ✓ | 🗶 | Proxy used: Other country averages |  | ✓ | ✓ | ✓ | ✓ | ✓ | ✓ | ✓ | ✓ |
| Not included in LiST modelling | | | | | | | | | | | | |
| ✓: Data available  🗶: Data not available  *Only included in India, Nepal and Sri Lanka LiST models | | | | | | | | | | | | |

Table B: Estimated school dropout rates by child age, gender, and wealth quintile

| **Child characteristic** | **Primary school (7 - 12 years)** | **Secondary school (13 - 19 years)** |
| --- | --- | --- |
| Wealth quintile |  |  |
| 1st | 6.2 | 11.3 |
| 2nd | 2.5 | 4.5 |
| 3rd | 2.4 | 2.3 |
| 4th | 1 | 2.2 |
| 5th | 1 | 2.2 |
| Gender |  |  |
| Male | 2.4 | 5.6 |
| Female | 2.6 | 3.9 |
| Adapted from Frankenburg et al(1) | | |

Table C: Median years of education completed by age and gender in six South Asian countries

| **Country** | **DHS year** | **Age category** | **Gender** | **Median years of schooling*** | **Years of schooling lost** | |
| --- | --- | --- | --- | --- | --- | --- |
|  |  |  |  |  | **Primary** | **Secondary** |
| Afghanistan | 2015 | 20 – 24 years | Male | 7.3 | 4.8 | 0 |
| Bangladesh | 2014 | 15 – 19 years | Female | 8.3 | 5.8 | 0 |
| India | 2015 – 16 | 20 – 24 years | Male | 10 | 7.5 | 1.5 |
| Nepal | 2016 | 20 – 24 years | Male | 9.1 | 6.6 | 0.6 |
| Pakistan | 2017 – 18 | 20 – 24 years | Male | 7.7 | 5.2 | 0 |
| Sri Lanka | 2016 | 20 – 24 years | Female | 11.4 | 8.9 | 2.9 |
| Source: Most recent country DHS(2-7)  *Assumed for both boys and girls currently enrolled in school | | | | | | |

Table D: Observed coverage disruption of SRMNCH services due to the COVID-19 pandemic response in South Asia, Jan 2020 – Jun 2021

|  | **Afghanistan** | | | | | |
| --- | --- | --- | --- | --- | --- | --- |
| **Interventions** | **Change in 2020** | | | | **Change in 2021** | |
|  | **Q1** | **Q2** | **Q3** | **Q4** | **Q1** | **Q2** |
| FP clients | 7.3% | -17.9% | -18.2% | -6.8% | 145.0% | 115.0% |
| Injectables | 7.3% | -17.9% | -18.2% | -6.8% | 145.0% | 115.0% |
| Condom | 7.3% | -17.9% | -18.2% | -6.8% | 145.0% | 115.0% |
| IUD | 7.3% | -17.9% | -18.2% | -6.8% | 145.0% | 115.0% |
| Female sterilisation | 7.3% | -17.9% | -18.2% | -6.8% | 145.0% | 115.0% |
| Male sterilisation | 7.3% | -17.9% | -18.2% | -6.8% | 145.0% | 115.0% |
| Implant | 7.3% | -17.9% | -18.2% | -6.8% | 145.0% | 115.0% |
| Safe abortion services | -- | -- | -- | -- | -- | -- |
| Post abortion case management | -- | -- | -- | -- | -- | -- |
| Ectopic pregnancy case management | -- | -- | -- | -- | -- | -- |
| Antenatal care (4+ visits) | 0.1% | -14.4% | 2.2% | -2.8% | 9.0% | 4.6% |
| Tetanus Toxoid (2 or more doses) | 0.1% | -14.4% | 2.2% | -2.8% | 9.0% | 4.6% |
| Syphilis detection and treatment | -- | -- | -- | -- | -- | -- |
| Calcium supplementation | -- | -- | -- | -- | -- | -- |
| Iron supplementation in pregnancy | -- | -- | -- | -- | -- | -- |
| Hypertensive disorder case management | -- | -- | -- | -- | -- | -- |
| Diabetes case management | -- | -- | -- | -- | -- | -- |
| Malaria case management | -- | -- | -- | -- | -- | -- |
| MgSO4 management of pre-eclampsia | -- | -- | -- | -- | -- | -- |
| Fetal growth restriction detection and management | -- | -- | -- | -- | -- | -- |
| Total home deliveries by clinic staff | -43.1% | -32.2% | -29.1% | -22.5% | -52.9% | -42.9% |
| Facility births | 4.4% | -0.4% | -0.7% | 0.9% | 12.8% | 16.0% |
| Postnatal visit within 2 days after birth | 4.3% | -6.3% | -2.2% | -1.8% | 8.3% | 14.4% |
| Vitamin A supplementation | -- | -- | -- | -- | -- | -- |
| Zinc Supplementation | -- | -- | -- | -- | -- | -- |
| Vaccine: BCG | -- | -- | -- | -- | -- | -- |
| Vaccine:OPV3 | -- | -- | -- | -- | -- | -- |
| Vaccine:DPT3/Penta3 | 6.6% | -9.5% | 13.4% | 4.5% | 7.9% | 1.2% |
| Vaccine:HepB3 | -- | -- | -- | -- | -- | -- |
| Vaccine: PCV3 | -- | -- | -- | -- | -- | -- |
| Vaccine:RV2 | -- | -- | -- | -- | -- | -- |
| Vaccine: Measles | -4.5% | -7.0% | 6.7% | -0.9% | 4.9% | 5.8% |
| Fully immunization | -- | -- | -- | -- | -- | -- |
| Maternal sepsis case management | -- | -- | -- | -- | -- | -- |
| ORS - oral rehydration solution | -- | -- | -- | -- | -- | -- |
| Antibiotics for treatment of dysentery | -- | -- | -- | -- | -- | -- |
| Antibiotics for pneumonia | 15.2% | -8.6% | -0.4% | -2.7% | 5.5% | 7.9% |
| Vitamin A for treatment of measles | -- | -- | -- | -- | -- | -- |
| ACTs - Artemisinin compounds for treatment of malaria | -- | -- | -- | -- | -- | -- |
| SAM - treatment for severe acute malnutrition | 4.6% | -37.4% | -19.0% | -29.2% | 36.0% | 54.0% |
|  | **Bangladesh** | | | | | |
| **Interventions** | **Change in 2020** | | | | **Change in 2021** | |
|  | **Q1** | **Q2** | **Q3** | **Q4** | **Q1** | **Q2** |
| FP clients | -21.4% | -62.0% | -37.6% | -28.0% | -3.4% | -22.2% |
| Injectables | -12.2% | -33.9% | -15.2% | -12.5% | 5.2% | 3.0% |
| Condom | -32.9% | -30.0% | -15.3% | -16.0% | -15.2% | 6.4% |
| IUD | -2.5% | -58.3% | -34.4% | -38.0% | 13.1% | -14.2% |
| Female sterilisation | -53.9% | -51.6% | -43.1% | -25.8% | -12.5% | -18.5% |
| Male sterilisation | -49.1% | -88.6% | -58.9% | -41.4% | -58.4% | -58.4% |
| Implant | 10.6% | -77.5% | -36.4% | -22.2% | 35.9% | -22.9% |
| Safe abortion services | -- | -- | -- | -- | -- | -- |
| Post abortion case management | -- | -- | -- | -- | -- | -- |
| Ectopic pregnancy case management | -- | -- | -- | -- | -- | -- |
| Antenatal care (4+ visits) | 6.7% | -45.1% | -48.3% | -36.3% | -18.5% | -26.4% |
| Tetanus Toxoid (2 or more doses) | 6.7% | -45.1% | -48.3% | -36.3% | -18.5% | -26.4% |
| Syphilis detection and treatment | -- | -- | -- | -- | -- | -- |
| Calcium supplementation | -- | -- | -- | -- | -- | -- |
| Iron supplementation in pregnancy | -- | -- | -- | -- | -- | -- |
| Hypertensive disorder case management | -- | -- | -- | -- | -- | -- |
| Diabetes case management | -- | -- | -- | -- | -- | -- |
| Malaria case management | -- | -- | -- | -- | -- | -- |
| MgSO4 management of pre-eclampsia | -- | -- | -- | -- | -- | -- |
| Fetal growth restriction detection and management | -- | -- | -- | -- | -- | -- |
| Total home deliveries by clinic staff | -- | -- | -- | -- | -- | -- |
| Facility births | -7.0% | -42.0% | -29.8% | -15.5% | -13.3% | -27.2% |
| Postnatal visit within 2 days after birth | -14.4% | -43.2% | -39.0% | -17.6% | -66.0% | -74.9% |
| Vitamin A supplementation | -- | -- | -- | -- | -- | -- |
| Zinc Supplementation | -- | -- | -- | -- | -- | -- |
| Vaccine: BCG | -- | -- | -- | -- | -- | -- |
| Vaccine:OPV3 | -- | -- | -- | -- | -- | -- |
| Vaccine:DPT3/Penta3 | -6.0% | -36.7% | 20.1% | -1.5% | 9.2% | 5.9% |
| Vaccine:HepB3 | -- | -- | -- | -- | -- | -- |
| Vaccine: PCV3 | -- | -- | -- | -- | -- | -- |
| Vaccine:RV2 | -- | -- | -- | -- | -- | -- |
| Vaccine: Measles | -6.3% | -18.5% | 7.9% | -12.7% | 104.4% | 113.8% |
| Fully immunization | -- | -- | -- | -- | -- | -- |
| Maternal sepsis case management | -- | -- | -- | -- | -- | -- |
| ORS - oral rehydration solution | -- | -- | -- | -- | -- | -- |
| Antibiotics for treatment of dysentery | -- | -- | -- | -- | -- | -- |
| Antibiotics for pneumonia | -13.2% | -71.3% | -55.2% | -27.5% | -3.9% | -23.7% |
| Vitamin A for treatment of measles | -- | -- | -- | -- | -- | -- |
| ACTs - Artemisinin compounds for treatment of malaria | -- | -- | -- | -- | -- | -- |
| SAM - treatment for severe acute malnutrition | -27.0% | -70.5% | -27.2% | 59.3% | 103.0% | 100.7% |
|  | **India** | | | | | |
| **Interventions** | **Change in 2020** | | | | **Change in 2021** | |
|  | **Q1** | **Q2** | **Q3** | **Q4** | **Q1** | **Q2** |
| FP clients | -0.8% | -25.0% | -3.2% | 3.6% | 4.7% | -38.6% |
| Injectables | -10.2% | -64.3% | -38.6% | 10.1% | 21.0% | -58.8% |
| Condom | -1.6% | -25.4% | -4.4% | 2.8% | 3.5% | -39.5% |
| IUD | 1.2% | -49.5% | -21.3% | -4.7% | -0.2% | -53.7% |
| Female sterilisation | -1.1% | -86.3% | -54.0% | -15.7% | 9.3% | -81.4% |
| Male sterilisation | -4.8% | -86.2% | -79.3% | -43.6% | -16.9% | -85.2% |
| Implant | -- | -- | -- | -- | -- | -- |
| Safe abortion services | -5.6% | -43.2% | -35.6% | -25.6% | -17.3% | -59.1% |
| Post abortion case management | 119.7% | -5.5% | -9.4% | -4.8% | 118.7% | -27.9% |
| Ectopic pregnancy case management | 8.2% | -27.7% | -18.6% | -9.1% | -7.0% | -54.7% |
| Antenatal care (4+ visits) | 4.7% | -26.7% | -8.5% | -1.0% | 7.4% | -41.5% |
| Tetanus Toxoid (2 or more doses) | -2.2% | -25.3% | -5.1% | -3.3% | -1.1% | -38.4% |
| Syphilis detection and treatment | 2.7% | 16.6% | 23.9% | -8.3% | 199.4% | -5.5% |
| Calcium supplementation | 18.1% | -9.4% | 4.3% | 10.6% | 34.5% | -29.7% |
| Iron supplementation in pregnancy | -0.3% | -19.7% | -4.2% | 2.6% | 5.1% | -40.4% |
| Hypertensive disorder case management | 22.5% | -29.2% | -7.7% | -0.4% | 14.5% | -44.0% |
| Diabetes case management | 10.3% | -29.2% | -3.7% | 26.0% | 45.3% | 0.2% |
| Malaria case management | 4.7% | -26.7% | -8.5% | -1.0% | 7.4% | -41.5% |
| MgSO4 management of pre-eclampsia | 8.2% | -27.7% | -18.6% | -9.1% | -7.0% | -54.7% |
| Fetal growth restriction detection and management | 6.1% | -26.7% | 1.4% | 13.2% | 27.6% | -34.1% |
| Total home deliveries by clinic staff | -19.4% | -28.2% | -5.2% | -0.4% | -16.2% | -42.8% |
| Facility births | -2.1% | -28.2% | -7.5% | -2.6% | -0.7% | -46.7% |
| Postnatal visit within 2 days after birth | -17.9% | -24.9% | -33.3% | -22.2% | -23.2% | -55.8% |
| Vitamin A supplementation | -0.7% | -78.5% | -38.2% | 0.3% | -5.9% | -63.4% |
| Zinc Supplementation | -18.8% | -87.1% | -23.5% | -12.5% | -8.1% | -65.7% |
| Vaccine: BCG | -3.6% | -28.0% | -6.6% | -0.5% | -6.7% | -48.2% |
| Vaccine:OPV3 | 2.5% | -37.0% | 0.6% | 1.6% | 3.9% | -44.2% |
| Vaccine:DPT3/Penta3 | 2.8% | -37.0% | 1.0% | 1.9% | 4.8% | -44.0% |
| Vaccine:HepB3 | -11.2% | 38.9% | 146.4% | 184.2% | 121.0% | 40.6% |
| Vaccine: PCV3 | -3.9% | -27.6% | 1.0% | 1.9% | 7.3% | -44.5% |
| Vaccine:RV2 | 94.6% | 28.6% | 55.5% | 13.6% | 104.8% | 9.9% |
| Vaccine: Measles | -67.8% | -54.6% | -31.0% | -24.2% | -64.6% | -62.9% |
| Fully immunization | -- | -- | -- | -- | -- | -- |
| Maternal sepsis case management | 8.2% | -27.7% | -18.6% | -9.1% | -7.0% | -54.7% |
| ORS - oral rehydration solution | -22.0% | -79.0% | 8.8% | -16.1% | 29.3% | -71.1% |
| Antibiotics for treatment of dysentery | -8.8% | -74.6% | -70.3% | -53.3% | -32.1% | -74.5% |
| Antibiotics for pneumonia | 27.4% | -29.9% | -9.4% | -46.6% | 131.3% | -67.9% |
| Vitamin A for treatment of measles | 63.6% | -40.3% | 2.8% | 33.6% | 55.7% | -38.2% |
| ACTs - Artemisinin compounds for treatment of malaria | 0.9% | -87.8% | -67.6% | -35.6% | -8.2% | -87.3% |
| SAM - treatment for severe acute malnutrition | 5.1% | -48.2% | -14.9% | 9.6% | 18.4% | -26.7% |
|  | **Nepal** | | | | | |
| **Interventions** | **Change in 2020** | | | | **Change in 2021** | |
|  | **Q1** | **Q2** | **Q3** | **Q4** | **Q1** | **Q2** |
| FP clients | -22.3% | -51.9% | 57.9% | -5.3% | -1.3% | -42.9% |
| Injectables | -22.3% | -51.9% | 57.9% | -5.3% | -1.3% | -42.9% |
| Condom | -19.5% | -51.8% | -3.6% | -1.8% | -9.2% | -28.2% |
| IUD | -35.0% | -60.8% | 119.3% | -20.5% | -12.5% | -64.0% |
| Female sterilisation | -22.3% | -51.9% | 57.9% | -5.3% | -1.3% | -42.9% |
| Male sterilisation | -22.3% | -51.9% | 57.9% | -5.3% | -1.3% | -42.9% |
| Implant | -12.6% | -43.1% | 58.1% | 6.6% | 17.9% | -36.4% |
| Safe abortion services | -- | -- | -- | -- | -- | -- |
| Post abortion case management | -- | -- | -- | -- | -- | -- |
| Ectopic pregnancy case management | -- | -- | -- | -- | -- | -- |
| Antenatal care (4+ visits) | -9.3% | -29.2% | -14.7% | -5.6% | 20.5% | -15.4% |
| Tetanus Toxoid (2 or more doses) | -9.3% | -29.2% | -14.7% | -5.6% | 20.5% | -15.4% |
| Syphilis detection and treatment | -- | -- | -- | -- | -- | -- |
| Calcium supplementation | -- | -- | -- | -- | -- | -- |
| Iron supplementation in pregnancy | -13.6% | -26.2% | -6.8% | -1.1% | -3.1% | -17.8% |
| Hypertensive disorder case management | -- | -- | -- | -- | -- | -- |
| Diabetes case management | -- | -- | -- | -- | -- | -- |
| Malaria case management | -- | -- | -- | -- | -- | -- |
| MgSO4 management of pre-eclampsia | -- | -- | -- | -- | -- | -- |
| Fetal growth restriction detection and management | -- | -- | -- | -- | -- | -- |
| Total home deliveries by clinic staff | -- | -- | -- | -- | -- | -- |
| Facility births | -6.6% | -26.9% | -8.3% | -4.9% | 2.5% | -13.1% |
| Postnatal visit within 2 days after birth | -6.6% | -15.2% | 15.7% | 31.5% | -4.4% | -13.6% |
| Vitamin A supplementation | -91.4% | -2.5% | -65.3% | 5.8% | -55.2% | -10.5% |
| Zinc Supplementation | -- | -- | -- | -- | -- | -- |
| Vaccine: BCG | -- | -- | -- | -- | -- | -- |
| Vaccine:OPV3 | -- | -- | -- | -- | -- | -- |
| Vaccine:DPT3/Penta3 | -22.7% | 3.0% | 19.7% | 1.5% | 6.3% | -6.0% |
| Vaccine:HepB3 | -- | -- | -- | -- | -- | -- |
| Vaccine: PCV3 | -- | -- | -- | -- | -- | -- |
| Vaccine:RV2 | -- | -- | -- | -- | -- | -- |
| Vaccine: Measles | -23.7% | -8.7% | 20.9% | 6.5% | -74.9% | -72.8% |
| Fully immunization | -- | -- | -- | -- | -- | -- |
| Maternal sepsis case management | -- | -- | -- | -- | -- | -- |
| ORS - oral rehydration solution | -- | -- | -- | -- | -- | -- |
| Antibiotics for treatment of dysentery | -- | -- | -- | -- | -- | -- |
| Antibiotics for pneumonia | -11.5% | -48.7% | -54.7% | -56.3% | -37.6% | -53.7% |
| Vitamin A for treatment of measles | -- | -- | -- | -- | -- | -- |
| ACTs - Artemisinin compounds for treatment of malaria | -- | -- | -- | -- | -- | -- |
| SAM - treatment for severe acute malnutrition | -53.1% | -83.4% | -53.2% | -34.0% | 30.8% | 27.0% |
|  | **Pakistan** | | | | | |
| **Interventions** | **Change in 2020** | | | | **Change in 2021** | |
|  | **Q1** | **Q2** | **Q3** | **Q4** | **Q1** | **Q2** |
| FP clients | -31.8% | -71.7% | -35.4% | -14.1% | -22.8% | -33.1% |
| Injectables | -25.5% | -62.7% | -24.5% | -3.7% | -19.7% | -23.8% |
| Condom | -22.9% | -85.3% | -43.8% | -7.4% | -21.6% | -43.1% |
| IUD | -23.5% | -75.6% | -27.0% | -23.2% | -10.5% | -65.6% |
| Female sterilisation | -27.1% | -86.4% | -75.9% | -84.4% | -83.4% | -85.9% |
| Male sterilisation | -86.7% | -98.6% | -93.3% | -99.3% | -100.0% | -98.4% |
| Implant | -63.0% | -85.0% | -56.3% | -26.6% | -64.3% | -75.8% |
| Safe abortion services | -- | -- | -- | -- | -- | -- |
| Post abortion case management | 5.1% | -47.7% | -34.4% | 2.3% | -0.1% | -7.9% |
| Ectopic pregnancy case management | -- | -- | -- | -- | -- | -- |
| Antenatal care (4+ visits) | -16.7% | -56.2% | -27.9% | -4.7% | -28.7% | -29.7% |
| Tetanus Toxoid (2 or more doses) | -14.8% | -40.5% | -29.1% | -18.9% | -31.3% | -19.7% |
| Syphilis detection and treatment | -- | -- | -- | -- | -- | -- |
| Calcium supplementation | -- | -- | -- | -- | -- | -- |
| Iron supplementation in pregnancy | -- | -- | -- | -- | -- | -- |
| Hypertensive disorder case management | -- | -- | -- | -- | -- | -- |
| Diabetes case management | -- | -- | -- | -- | -- | -- |
| Malaria case management | -- | -- | -- | -- | -- | -- |
| MgSO4 management of pre-eclampsia | -- | -- | -- | -- | -- | -- |
| Fetal growth restriction detection and management | -- | -- | -- | -- | -- | -- |
| Total home deliveries by clinic staff | -- | -- | -- | -- | -- | -- |
| Facility births | 0.0% | -36.7% | -24.9% | -3.3% | -14.5% | -28.8% |
| Postnatal visit within 2 days after birth | 4.7% | -48.1% | -27.4% | -7.7% | -20.3% | -34.4% |
| Vitamin A supplementation | -- | -- | -- | -- | -- | -- |
| Zinc Supplementation | -- | -- | -- | -- | -- | -- |
| Vaccine: BCG | -- | -- | -- | -- | -- | -- |
| Vaccine:OPV3 | -- | -- | -- | -- | -- | -- |
| Vaccine:DPT3/Penta3 | -22.4% | -55.1% | -26.7% | -10.2% | -28.8% | -22.1% |
| Vaccine:HepB3 | -- | -- | -- | -- | -- | -- |
| Vaccine: PCV3 | -- | -- | -- | -- | -- | -- |
| Vaccine:RV2 | -- | -- | -- | -- | -- | -- |
| Vaccine: Measles | -11.6% | -40.8% | -25.9% | -17.7% | -30.4% | -17.9% |
| Fully immunization | -4.3% | -37.2% | -23.8% | -28.0% | -34.7% | -24.2% |
| Maternal sepsis case management | -- | -- | -- | -- | -- | -- |
| ORS - oral rehydration solution | -- | -- | -- | -- | -- | -- |
| Antibiotics for treatment of dysentery | -- | -- | -- | -- | -- | -- |
| Antibiotics for pneumonia | -10.3% | -69.0% | -43.4% | -29.5% | -38.5% | -41.7% |
| Vitamin A for treatment of measles | -- | -- | -- | -- | -- | -- |
| ACTs - Artemisinin compounds for treatment of malaria | -- | -- | -- | -- | -- | -- |
| SAM - treatment for severe acute malnutrition | 32.2% | -60.8% | -37.0% | -8.1% | -34.2% | -43.6% |
|  | **Sri Lanka** | | | | | |
| **Interventions** | **Change in 2020** | | | | **Change in 2021** | |
|  | **Q1** | **Q2** | **Q3** | **Q4** | **Q1** | **Q2** |
| FP clients | -17.3% | -6.6% | 18.7% | 7.1% | 2.5% | -17.2% |
| Injectables | -22.4% | -11.9% | -4.6% | 0.9% | -5.9% | -14.0% |
| Condom | -11.1% | 3.4% | 23.8% | 11.0% | 8.8% | -13.0% |
| IUD | -30.4% | -33.7% | 12.7% | -4.9% | -14.1% | -35.5% |
| Female sterilisation | -1.4% | -7.7% | 26.0% | 13.2% | 16.7% | -10.0% |
| Male sterilisation | -21.1% | -54.5% | -31.3% | -50.0% | -42.1% | 54.5% |
| Implant | -18.9% | -9.0% | 43.0% | 20.2% | 12.3% | -19.8% |
| Safe abortion services | -- | -- | -- | -- | -- | -- |
| Post abortion case management | -- | -- | -- | -- | -- | -- |
| Ectopic pregnancy case management | -- | -- | -- | -- | -- | -- |
| Antenatal care (4+ visits) | 0.0% | -2.1% | -2.5% | -1.9% | -2.3% | -2.6% |
| Tetanus Toxoid (2 or more doses) | 0.0% | -2.1% | -2.5% | -1.9% | -2.3% | -2.6% |
| Syphilis detection and treatment | -- | -- | -- | -- | -- | -- |
| Calcium supplementation | -- | -- | -- | -- | -- | -- |
| Iron supplementation in pregnancy | -- | -- | -- | -- | -- | -- |
| Hypertensive disorder case management | -- | -- | -- | -- | -- | -- |
| Diabetes case management | -- | -- | -- | -- | -- | -- |
| Malaria case management | -- | -- | -- | -- | -- | -- |
| MgSO4 management of pre-eclampsia | -- | -- | -- | -- | -- | -- |
| Fetal growth restriction detection and management | -- | -- | -- | -- | -- | -- |
| Total home deliveries by clinic staff | -- | -- | -- | -- | -- | -- |
| Facility births | 0.0% | 0.0% | 0.0% | 0.0% | 0.0% | 0.0% |
| Postnatal visit within 2 days after birth | -6.1% | -1.8% | 1.9% | -0.1% | -10.9% | -7.1% |
| Vitamin A supplementation | -18.8% | 2.9% | 6.3% | 4.5% | 15.4% | 4.8% |
| Zinc Supplementation | -- | -- | -- | -- | -- | -- |
| Vaccine: BCG | -- | -- | -- | -- | -- | -- |
| Vaccine:OPV3 | -- | -- | -- | -- | -- | -- |
| Vaccine:DPT3/Penta3 | -11.1% | -24.6% | 6.7% | -1.4% | -1.4% | -5.2% |
| Vaccine:HepB3 | -- | -- | -- | -- | -- | -- |
| Vaccine: PCV3 | -- | -- | -- | -- | -- | -- |
| Vaccine:RV2 | -- | -- | -- | -- | -- | -- |
| Vaccine: Measles | -11.6% | -18.8% | 2.4% | -6.2% | 1.0% | 7.3% |
| Fully immunization | -- | -- | -- | -- | -- | -- |
| Maternal sepsis case management | -- | -- | -- | -- | -- | -- |
| ORS - oral rehydration solution | -- | -- | -- | -- | -- | -- |
| Antibiotics for treatment of dysentery | -- | -- | -- | -- | -- | -- |
| Antibiotics for pneumonia | -5.0% | -49.4% | -38.4% | -29.0% | -18.6% | -27.8% |
| Vitamin A for treatment of measles | -- | -- | -- | -- | -- | -- |
| ACTs - Artemisinin compounds for treatment of malaria | -- | -- | -- | -- | -- | -- |
| SAM - treatment for severe acute malnutrition | -10.8% | -63.0% | -34.1% | -3.0% | 30.8% | 27.0% |
| Based on actual health systems data  Approximated using related indicators for same country  Approximated using related indicators from other countries  -: Data not received | | | | | | |

Table E: Observed coverage disruption of selected SRMNCH services due to COVID-19 pandemic restrictions in Pakistan, January 2020 to June 2021

|  | **Change in 2020** | | | | **Change in 2021** | |
| --- | --- | --- | --- | --- | --- | --- |
|  | **Q1, median (95% CI)** | **Q2, median (95% CI)** | **Q3, median (95% CI)** | **Q4, median (95% CI)** | **Q1, median (95% CI)** | **Q2, median (95% CI)** |
| FP services | -29.4% (-35.3%, -21.6%) | -72.0% (-77.5%, -61.6%) | -39.5% (-46.3%, -31.3%) | -13.3% (-18.7%, -7.2%) | -22.2% (-32.8%, -15.3%) | -30.5% (-39.5%, -23.9%) |
| 4+ ANC visits | -12.3% (-15.8%, -6.6%) | -53.3% (-57.5%, -48.7%) | -26.2% (-31.9%, -19.4%) | -7.4% (-12.3%, -3.0%) | -22.3% (-26.4%, -18.3%) | -21.6% (-27.2%, -17.5%) |
| Facility births | 2.2% (0.0%, 5.8%) | -29.9% (-35.7%, -25.3%) | -17.9% (-20.9%, -13.3%) | 0.0% (-3.8%, 2.2%) | -4.4% (-8.1%, -1.1%) | -20.0% (-24.7%, -13.4%) |
| Vaccine: DPT3/Penta3 | -11.9% (-15.9%, -8.1%) | -46.1% (-51.9%, -41.2%) | -9.8% (-14.0%, -5.1%) | 2.9% (-2.8%, 11.3%) | -15.6% (-19.6%, -10.9%) | -21.4% (-26.4%, -17.0%) |
| Antibiotics for pneumonia | -19.7% (-33.3%, -7.0%) | -76.6% (-80.9%, -70.6%) | -45.5% (-54.5%, -38.4%) | -28.6% (-37.7%, -12.2%) | -35.9% (-48.6%, -22.0%) | -48.1% (-57.5%, -36.4%) |
| SAM treatment | -45.4% (-76.3%, -20.1%) | -69.6% (-100.0%, -56.6%) | -83.8% (-100.0%, -53.2%) | -75.8% (-98.3%, -35.2%) | -50.0% (-89.9%, -28.4%) | -90.6% (-100.0%, -56.7%) |
| Note: The point estimates are different from those presented in Table 1, as the % change are estimated using medians from DHIS data | | | | | | |

Table F: Estimated increase in maternal and child deaths and unintended pregnancies due to COVID-19 pandemic restrictions in Pakistan, January 2020 to June 2021

| **Quarter** | **Child Mortality (0-59 months)** | **Neonatal mortality (<1 month)** | **Stillbirths** | **Maternal Deaths** | **Unintended pregnancies** |
| --- | --- | --- | --- | --- | --- |
| 2020-Q1 | 2.7% (0.4%, 4.9%) | 1.4% (-0.8%, 3.3%) | 1.4% (-0.1%, 2.6%) | 2.2% (-0.5%, 4.6%) | 4.6% (0.3%, 8.5%) |
| 2020-Q2 | 17.5% (15.4%, 19.8%) | 17.6% (15.3%, 20.5%) | 9.9% (8.8%, 11.1%) | 18.8% (16.5%, 21.5%) | 21.3% (19.6%, 23.1%) |
| 2020-Q3 | 9.7% (7.5%, 11.4%) | 10.6% (8.1%, 12.3%) | 6.3% (5%, 7.2%) | 11.6% (8.8%, 13.5%) | 14.1% (11.2%, 15.8%) |
| 2020-Q4 | 4% (1.4%, 5.8%) | 3.6% (1.6%, 5.4%) | 3.2% (1.7%, 4.1%) | 4.7% (2.3%, 6.9%) | 11.2% (6.4%, 12.9%) |
| 2021-Q1 | 4.3% (4.0%, 4.5%) | 4.7% (4.5%, 5.4%) | 0.6% (0.3%, 1.5%) | 2.1% (1.9%, 2.8%) | 3.4% (3.3%, 5.4%) |
| 2021-Q2 | 8.3% (8.0%, 8.6%) | 9.9% (9.9%, 10.2%) | 2.8% (2.7%, 3.6%) | 6.6% (6.3%, 6.8%) | 4.7% (4.6%, 7.5%) |
| **Total** | **7.7% (6.3%, 9.2%)** | **8.0% (6.6%, 9.4%)** | **4.0% (3.4%, 4.7%)** | **7.7% (6.1%, 9.1%)** | **9.9% (8.4%, 11.4%)** |
| Note: The point estimates are different from the table 2 as the coverages are estimated using median% from HMIS data | | | | | |

Table G: Number of estimated additional dropouts and present value income loss in six South Asian countries

| **Country** | **Number of dropouts** | | | **Income loss*** |
| --- | --- | --- | --- | --- |
|  | Primary school-aged | Secondary-school aged | Total |  |
| Afghanistan | 329,186 | 83,630 | 732,881 | 14.8% |
| Bangladesh | 332,862 | 359,972 | 692,834 | 16.9% |
| India | 2,513,686 | 4,986,212 | 7,499,899 | 23.9% |
| Nepal | 59,356 | 128,893 | 188,249 | 20.0% |
| Pakistan | 95,573 | 83,630 | 179,203 | 15.6% |
| Sri Lanka | 43,554 | 101,388 | 144,942 | 29.9% |
| **Total** | **3,374,218** | **6,063,789** | **9,438,007** | 20.2% |

Table H: Proportion of children from households in lowest, second and top 3wealth quintile, among those will permanently dropout of school in six South Asian countries

| **Country** | **School dropouts by wealth quintile** | | |
| --- | --- | --- | --- |
|  | Lowest | Second | Top 3 |
| Afghanistan | 44.8% | 18.2% | 37.0% |
| Bangladesh | 43.2% | 20.0% | 36.8% |
| India | 44.5% | 20.3% | 35.1% |
| Nepal | 49.7% | 19.1% | 31.2% |
| Pakistan | 29.2% | 20.0% | 50.8% |
| Sri Lanka | 49.0% | 19.7% | 31.4% |

# References

1. Frankenburg E, Thomas D, Beegle K. The real cost of Indonesia's economic crises: preliminary findings from the Indonesia Family Life Surveys. 1999.

2. Bangladesh Demographic and Health Survey 2014. Dhaka, Bangladesh, and Rockville, Maryland, USA: National Institute of Population Research and Training (NIPORT), Mitra and Associates, and ICF International; 2016.

3. National Family Health Survey (NFHS-4), 2015-16: India. Mumbai: International Institute for Population Sciences and ICF; 2017.

4. Nepal Demographic and Health Survey 2016. Kathmandu, Nepal: Ministry of Health, Nepal; New ERA; and ICF; 2017.

5. Afghanistan Demographic and Health Survey 2015. Kabul, Afghanistan: Central Statistics Organization (CSO), Ministry of Public Health (MoPH), and ICF; 2017.

6. Sri Lanka Demographic and Health Survey 2016. Sri Lanka: Department of Census and Statistics (DCS) and Ministry of Health, Nutrition and Indigenous Medicine; 2017.

7. Pakistan Demographic and Health Survey 2017-18. Islamabad, Pakistan, and Rockville, Maryland, USA: National Institute of Population Studies (NIPS) [Pakistan] and ICF; 2019.
